# Epigenetic regulation of the human telomerase reverse transciptase gene: A potential therapeutic target for the treatment of leukemia (Review)

**DOI:** 10.3892/ol.2013.1367

**Published:** 2013-05-29

**Authors:** XINBING SUI, NA KONG, ZHANGGUI WANG, HONGMING PAN

**Affiliations:** 1 Department of Medical Oncology, Sir Run Run Shaw Hospital, Zhejiang University, P.R. China; 2 Biomedical Research Center and Key Laboratory of Biotherapy, Hangzhou, Zhejiang 310016, P.R. China

**Keywords:** human telomerase reverse transcriptase, epigenetic, leukemia

## Abstract

Telomerase activation is a critical step in human carcinogenesis through the maintenance of telomeres. Telomerase activity is primarily regulated by the human telomerase reverse transcriptase gene (*hTERT*), thus, an improved understanding of the transcriptional control of *hTERT* may provide potential therapeutic targets for the treatment of leukemia and other forms of cancer. Epigenetic modulation, a significant regulatory process in cell biology, has recently been shown to be involved in the regulation of the *hTERT* gene. Moreover, several epigenetic modifiers, including DNA methyltransferase (DNMT) and histone deacetylase (HDAC) inhibitors, are now in pre- and early clinical trials of leukemia as monotherapies or in combination with other drugs, and have achieved significant clinical success. In the present review, the epigenetic mechanisms associated with telomerase activity in leukemia, and the therapeutic potential of an antitelomerase strategy that combines epigenetic modifiers with telomerase hTR subunit small molecule inhibitors are discussed.

## Contents

1. Introduction
2. Epigenetic regulation of *hTERT* and telomere length
3. Targeting telomerase *(hTERT)* in leukemia cells through epigenetic modifiers presents new anticancer therapeutic approaches for leukemia
4. Future perspectives

## 1. Introduction

Telomeres serve as essential structures that protect the ends of linear chromosomes from DNA repair and degradation, and their maintenance is critical for long-term cell proliferation and survival (1,2). Mammalian telomeres consist of tandem TTAGGG repeats that are bound by a specialized six-protein complex known as shelterin and may be replenished by telomerase (3). Telomerase is composed of two essential components, a catalytic subunit with reverse transcriptase activity, telomerase reverse transcriptase (TERT), and a telomerase RNA component (TERC), that acts as a template for DNA synthesis (4–6). Telomerase activity is overexpressed in the majority of cancer cells but is barely detectable in the predominance of normal somatic cells (7).

Among the various aspects of gene control, epigenetic alterations have gained attention as critical determinants for tumor initiation and subsequent cancer progression (8,9). The forms of epigenetic control of gene expression include DNA methylation and histone modification. DNA methylation involves a covalent modification at the fifth carbon position of cytosine residues within CpG dinucleotides, resulting in the transcriptional silencing of the affiliated gene (10). Promoter hypermethylation of tumor suppressor genes has been increasingly considered as a fundamental mechanism for the silencing of these genes in cancer cells, resulting in tumor initiation and progression (11,12). In addition to DNA methylation, another key element in the epigenetic control of gene expression is histone modification, including acetylation, methylation, phosphorylation and ubiquitination. Aberrant patterns of histone modifications have been associated with a large number of human malignancies (13,14). DNA methylation and histone modifications have been extensively recognized as epigenetic mechanisms that regulate gene transcription in carcinogenesis.

Human (*h)TERT*, a catalytic subunit of telomerase, is a key determinant for the control of telomerase activity (15). The *hTERT* promoter contains two E-box regions and five GC boxes (16). Similar to numerous human genes, *hTERT* also contains a CpG island in its promoter region, indicating a role for methylation in the regulation of *hTERT* expression (17). Accumulating evidence indicates that *hTERT* contains an increased level of DNA methylation in its promoter region in numerous cancers. Moreover, *hTERT* hypermethylation has been associated with the stable silencing of *hTERT* promoter expression (18,19). Histone deacetylation/methylation has also been reported to be responsible for the repressive status of the *hTERT* promoter (20). In the present review, the contribution of the epigenetic dysregulation of *hTERT* expression to leukemogenesis, and the prospect of this regulation as a basis for developing new anticancer therapies for leukemia are discussed.

## 2. Epigenetic regulation of *hTERT* and telomere length

Telomere length, maintained by telomerase, is a prominent mechanism for long-term cell proliferation and survival, and is strongly involved in cancer, cell senescence and aging (21–23). It has been demonstrated that the epigenetic plasticity of the *hTERT* gene promoter is a determinant for the control of telomerase activity. Therefore, inhibiting the expression of the *hTERT* gene through epigenetic mechanisms usually results in telomeric attrition. The epigenetic changes associated with the inhibition of telomerase activity include hypermethylation and histone modifications of the *hTERT* promoter.

**Figure 1.**
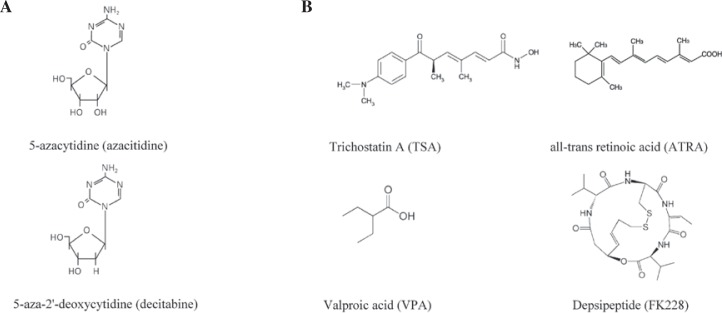
Chemical structures of selected (A) DNMT inhibitors and (B) HDAC inhibitors. DNMT, DNA methyltransferase; HDAC, histone deactylase.

**Figure 2.**
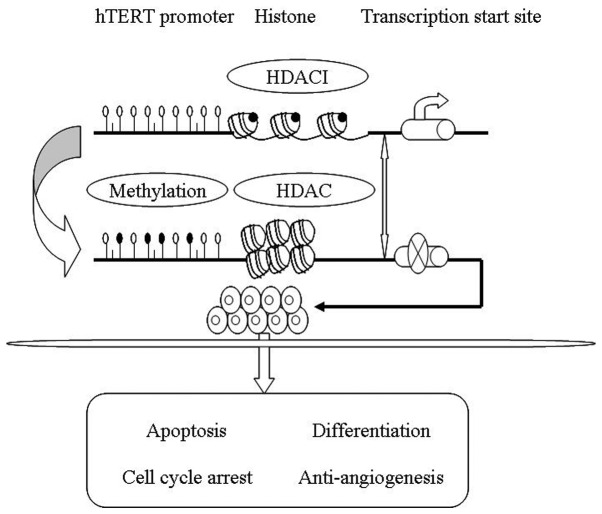
Complex molecular mechanisms and biological effects of *hTERT*. Epigenetic modification may affect *hTERT* expression and will form a permissive or inhibitive condition for *hTERT* transcription, depending on the specific cellular context. The suppression of *hTERT* promotes growth inhibition, differentiation, apoptosis and anti-angiogenesis. *hTERT,* human telomerase reverse transcriptase; HDAC, histone deacetylase; HDACI, HDAC inhibitor.

The proximal core promoter region of the *hTERT* gene harbors a high GC content and therefore, may be partly regulated by DNA methylation. Currently, there are three major DNA methyltransferases (DNMTs) identified to be responsible for the establishment of DNA methylation in the *hTERT* promoter (24). In the majority of cases, the aberrant methylation of CpG islands in promoter regions results in the heritable silencing of genes without a change in their coding sequence (25). Recent studies have shown that telomerase activity is repressed through the epigenetic silencing of *hTERT*, which is accompanied by telomere shortening (26,27). Shin *et al* reported that hypermethylation of the *hTERT* promoter played a critical role in the negative regulation of telomerase activity in normal human oral cells (27). Zinn *et al* also showed that the DNA methylation patterns of the *hTERT* promoter decreased *hTERT* transcription and telomerase activity, which was consistent with the normal paradigm of methylation-induced gene silencing (28). Paradoxically, there are conflicting studies with regard to the correlation between hypermethylation of the *hTERT* promoter, *hTERT* gene expression and telomerase activity. It is increasingly apparent that the *hTERT* promoter is partially or completely hyper-methylated in telomerase-positive tumors, but unmethylated or hypomethylated in telomerase-negative normal tissues (16,29). Treatment using 5-azacytidine (azacitidine) and its deoxy analogue 5-aza-2′-deoxycytidine (decitabine; DAC), two common demethylating agents, is able to cause a reduction in *hTERT* gene expression and consequently, telomerase activity (Fig. 1) (30–32). This correlation was in contrast with the general model of gene regulation by promoter methylation. Taken together, these studies indicate that *hTERT* may have an effect on telomerase activity through epigenetic regulation. However, the exact mechanism by which DNA methylation affects *hTERT* gene expression and telomerase activity remains to be elucidated (Fig. 2).

In addition to DNA methylation, another prevalent epigenetic mechanism that affects *hTERT* transcription is histone modification, including histone acetylation, methylation, phosphorylation and ubiquitinization. Histone tails carry basic charges and are associated with DNA molecules by electrostatic attraction. The acetylation of the histone proteins neutralizes the charge status of the histone tails, which decreases the attraction force between DNA and the histone tails, thus conferring an opened chromatin structure, allowing transcription factors, including c-MYC, MAD1 and CTCF, to bind to the DNA. Conversely, the deacetylation of histones results in the transcription factors having less access to the DNA (33,34). It has been demonstrated that Trichostatin A (TSA), a histone deacetylase (HDAC) inhibitor, is able to induce *hTERT* transcription and telomerase activity in normal cells and telomerase-negative immortal cell lines through the inhibition of histone deacetylation (Fig. 1) (35,36). Furthermore, FR901288, a novel cyclic peptide inhibitor of HDAC, has also been shown to activate *hTERT* mRNA expression in oral cancer cell lines (37). However, there are conflicting studies with regard to *hTERT* transcription and telomerase activity in cancer cells induced by HDAC inhibitors. Zhu *et al* reported that HDAC inhibitors prevented cell proliferation and induced apoptosis, but had no effect on the expression of hTERC and *hTERT* mRNA, or on telomerase activity (38). In prostate and brain cancer cells, the *hTERT* gene expression and telomerase activity were inhibited by HDAC inhibitors (30,40). Therefore, the HDAC inhibitors may exhibit various effects on *hTERT* transcription and telomerase activity in cancer cells. In addition to histone acetylation, *hTERT* transcription was also reported to be associated with histone methylation, of which three varying forms, including mono-, di- and trimethylation, may emerge in methylation modifications of the histone lysine residues. It has been demonstrated that mono- and dimethylated histone3-lysine9 (H3-K9) are localized to distinct domains of silent chromatin, where they are associated with inactive genes, whereas trimethylated H3-K9 is enriched in pericentric heterochromatin (41). Further studies have shown that a lack of *hTERT* expression in telomerase-negative cell lines is associated with histone H3 and H4 hypoacetylation and the methylation of H3-K9. However, *hTERT* transcription in telomerase-positive cell lines is associated with the hyperacetylation of H3 and H4 and the methylation of Lys4-H3 (H3-K4) (42). Histone methyltransferase (HMTase) is considered to be responsible for histone methylation at the *hTERT* promoter. Liu *et al* reported that SET and MYND domain-containing protein 3 (SMYD3), a HMTase, may directly transactivate *hTERT* transcription and telomerase activity in normal human fibroblasts and cancer cell lines through histone H3-K4 trimethylation (43). These results suggest that the epigenetic regulation of histones may contribute to *hTERT* gene expression and telomerase activity (Fig. 2).

## 3. Targeting telomerase *(hTERT)* in leukemia cells through epigenetic modifiers presents new anticancer therapeutic approaches for leukemia

Telomerase activity is a hallmark of the immortal cell phenotype and several mechanisms have been reported to be involved in its regulation, including transcriptional factors, DNA methylation and histone deacetylation. Furthermore, it has been shown that cells in numerous types of leukemia are able to maintain their telomere length and prevent replicative senescence or apoptosis by the epigenetic regulation of *hTERT* (44–46). Therefore, telomerase suppression using epigenetic modifications should be a promising target for the treatment of leukemia.

Studies have linked differentiation therapy to the epigenetic regulation of *hTERT*, and a large number of demethylating agents and HDAC inhibitors have achieved significant clinical successes in inducing the differentiation of human leukemia cells (Table I). Low methylation levels of the *hTERT* promoter core domain have been shown to correlate with high telomerase activity in patients with B-cell chronic lymphocytic leukemia (B-CLL), whereas a high degree of methylation indicates low enzyme activity. Moreover, patients with a high level of telomerase activity show a worse prognosis (47,48). Azacitidine and its deoxy analogue, decitabine, which are two DNMT inhibitors, have been approved as single agents to treat patients with leukemia through the induction of cell differentiation (Fig. 1) (49–52). HDAC inhibitors are agents that have attracted interest due to their ability to induce the differentiation of leukemic cells, and are now in pre- and early clinical trials as monotherapies and in combination with other drugs (53,54). Previous studies have shown that the transcriptional suppression of the *hTERT* gene during all-trans retinoic acid (ATRA) treatment is associated with the differentiation of leukemia cells, partly due to DNA methylation and histone deacetylation in the *hTERT* promoter region (47,55–56). Recently, it has been revealed that *hTERT* is downregulated 5-fold through epigenetic and protein acetylation mechanisms using a combined treatment of aurora kinase inhibitors (AKi) and HDAC inhibitors (57,58). Azouz *et al* identified two distinct functional domains of the *hTERT* promoter, the proximal and distal domains, and identified that the epigenetic modifications of the distal region determined the retinoid capacity to repress telomerase in maturation-resistant acute promyelocytic leukemia cells during cellular differentiation (59). Love *et al* showed that epigenetic regulation stabilized *hTERT* inhibition and thus maintained telomerase activity in a silenced state during the ATRA-induced differentiation of HL60 human leukemia cells (60). Altogether, these data indicate that epigenetic mechanisms may represent a target for maintaining the differentiated phenotype of human leukemia cells.

In addition to inducing cell differentiation, telomerase inhibition through epigenetic mechanisms has been reported to promote growth arrest, apoptosis and sensitivity to certain chemotherapeutic reagents in human acute leukemia cells. Woo *et al* demonstrated that TSA had an antiproliferative and apoptosis-inducing effect on the human leukemic cell line U937, and that these growth-inhibitory effects were associated with the inhibition of *hTERT* expression and telomerase activity. Therefore, a loss of telomerase activity may be a good surrogate biomarker to assess the antitumor activity of TSA in human leukemic cells (61). The resistance to imatinib is a major problem in chronic myelogenous leukemia (CML) treatment, and recent studies have shown that by targeting telomerase expression using a dominant-negative form of the catalytic protein subunit of *hTERT,* or by the treatment with HDAC inhibitors, the risk of imatinib resistance may be reduced and the imatinib-induced apoptosis in leukemia cells may be enhanced, suggesting that antitelomerase strategies may be able to prevent, or at least delay the onset of such resistance (62,63).

## 4. Future perspectives

The *hTERT* gene is usually transcriptionally inactivated in differentiated cells, but is reactivated in the majority of leukemia cells. As previously discussed, accumulating evidence suggests that epigenetic changes in the *hTERT* promoter may be a prominent mechanism of telomerase activity control. Therefore, antitelomerase strategies using epigenetic mechanisms may represent a promising target for the treatment of leukemia. There are two major approaches in advanced clinical trials to target telomerase-positive leukemia cells. Firstly, the use of direct telomerase hTR subunit small molecule inhibitors, such as telomestatin (SOT-095), several of which are currently in preclinical trials for acute leukemia (64,65). The second approach involves using epigenetic modification drugs against the *hTERT* protein; these drugs are currently being used for, or have completed trials for the treatment of leukemia. At present, there is an increasing interest in using epigenetic modifiers as candidate chemotherapeutic agents in human leukemia.

**Figure 3.**
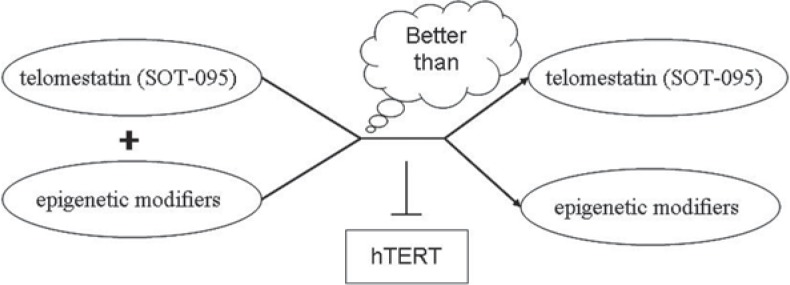
A hypothesis is associated with antitelomerase strategy. The antitelomerase strategy, created by combining epigenetic modifiers with telomerase hTR subunit small molecule inhibitors (such as SOT-095), may exert a more potent effect for the treatment of human leukemia, since each approach is able to individually inhibit telomerase activity. hTERT, human telomerase reverse transcriptase.

Epigenetic modifiers that are currently available in preclinical and early clinical trials of leukemia target DNMTs through DNMT inhibitors, or alter the status of the histones using HDAC inhibitors, in order to modulate gene transcription. It has been noted that epigenetic modifications contribute to *hTERT* gene expression and telomerase activity, resulting in a positive effect in the treatment of leukemia (59,61). In addition to epigenetic modifiers, the use of several telomerase hTR subunit small molecule inhibitors has resulted in the specific inhibition of telomerase activity. Therefore, an antitelomerase strategy involving a combination of epigenetic modifiers and telomerase hTR subunit small molecule inhibitors may exert a more potent effect for the treatment of human leukemia (Fig. 3).

Although epigenetic modifiers have shown promise as therapies for human leukemia in early clinical trials, certain limitations prevent their widespread clinical application. Firstly, the exact molecular mechanisms underlying the epigenetic regulation and *hTERT* expression remain to be elucidated, as do numerous details with regard to telomerase regulation. An improved understanding of the linkage will facilitate the identification of more specific and selective epigenetic modifiers for leukemia cells (66). Secondly, a broad spectrum of biological and potentially adverse effects have been identified following treatment using epigenetic modifiers. Further investigation with regard to these effects is required in large-scale and multicentric populations of treated patients (67). Thirdly, further studies will be required to identify whether the inhibition of *hTERT* gene expression is causal or consequential to the anticancer effects of epigenetic modifiers, and whether the *hTERT* gene or telomerase activity may be an appropriate predictive biomarker for assessing the antitumor activity of these agents in human leukemia cells (68). Finally, it should be taken into account whether the antitelomerase approach using epigenetic modifiers with telomerase hTR subunit small molecule inhibitors may be a better combinatorial strategy when compared with methods that are already used in prospective clinical trials.

Despite the unanswered biological questions, an increased understanding of the role of epigenetic regulation in *hTERT* gene expression and the treatment of leukemia may provide a prospective anticancer therapeutic approach in the form of the antitelomerase strategy.

## Figures and Tables

**Table I.** Selected drugs with epigenetic targets in the preclinical and clinical development of leukemia.

Drug target	Drug	Chemical class	Study in leukemia	Clinical trials
DNMT inhibitor	Azacitidine	Nucleoside analog	ALL, AML, CML	+
DNMT inhibitor	Decitabine (DAC)	Nucleoside analog	ALL, AML, CML	+
HDAC inhibitor	Valproic acid (VPA)	Short-chain fatty acid	AML, CLL, CML	+
HDAC inhibitor	Trichostatin A (TSA)	Hydroxamic acid	Preclinical trials	N/A
HDAC inhibitor	Panobinostat (LBH589)	Hydroxamic acid	ALL, AML	+
HDAC inhibitor	Depsipeptide (FR901228/FK228)	Cyclic tetrapeptide	AML	+
HDAC inhibitor	Entinostat (MS275/SNDX-275)	Benzamide	ALL, AML	+
HDAC inhibitor	MGCD0103	Benzamide	AML, CLL	+

AML, acute myelogenous leukemia; ALL, acute lymphocytic leukemia; CLL, chronic lymphocytic leukemia; CML, chronic myelogenous leukemia. DNMT, DNA methyltransferase; HDAC, histone deactylase.
